# Improved Dielectrically Modulated Quad Gate Schottky Barrier MOSFET Biosensor

**DOI:** 10.3390/mi14030685

**Published:** 2023-03-20

**Authors:** Papanasam Esakki, Prashanth Kumar, Manikandan Esakki, Adithya Venkatesh

**Affiliations:** 1 School of Electronics Engineering, Vellore Institute of Technology Chennai Campus, Chennai 600 127, India; 2 Centre for Innovation and Product Development (CIPD), Vellore Institute of Technology Chennai Campus, Chennai 600 127, India

**Keywords:** dielectric modulation, Schottky barrier, quad gate, biosensor, MOSFET

## Abstract

A novel Schottky barrier MOSFET with quad gate and with source engineering has been proposed in this work. A high-κ dielectric is used at the source side of the channel, while SiO_2_ is used at the drain side of the channel. To improve the carrier mobility, a SiGe pocket region is created at the source side of the channel. Physical and electrical characteristics of the proposed device are compared with conventional double gate Schottky barrier MOSFET. It has been observed that the proposed device exhibits better performance, with a higher I_ON_/I_OFF_ ratio and lower subthreshold slope. The high-κ dielectric, along with the SiGe pocket region, improves tunneling probability, while aluminum, along with SiO_2_ at the drain side, broadens the drain/channel Schottky barrier and reduces the hole tunneling probability, resulting in a reduced OFF-state current. Further, the proposed device is used as a biosensor to detect both the charged and neutral biomolecules. Biosensors are made by creating a nanocavity in the dielectric region near the source end of the channel to capture biomolecules. Biomolecules such as streptavidin, biotin, APTES, cellulose and DNA have unique dielectric constants, which modulates the electrical parameters of the device. Different electrical parameters, viz., the electric field, surface potential and drain current, are analyzed for each biomolecule. It has been observed that drain current increases with the dielectric constant of the biomolecules. Furthermore, the sensitivity and selectivity of the proposed biosensors is better than that of conventional biosensors made using double gate Schottky barrier MOSFETs. Sensitivity is almost twice that of a conventional sensor, while selectivity is six to twelve times higher than a conventional one.

## 1. Introduction

Accurate measurement of important physiological parameters facilitates the timely discovery of potential diseases that deteriorate the health of a patient. The necessity for a early and precise identification of ailments and other vital examinations of living organisms produced a growing need for economic, highly selective and sensitive biosensors. A biosensor is a device which transforms the biological properties of biomolecules into corresponding electrical characteristics. Factors such as inexpensive fabrication, quick response time, congeniality with modern state-of-the-art systems, smaller size and label-free detection give an edge to Field Effect Transistor (FET) based biosensors among the other semiconductor-based biosensors. Presently, FET biosensors are principally used in various industries such as food and beverage, medicines, agriculture and environmental monitoring. The FET-based biosensors work on the principle of modulating the electrical parameters of the device with the dielectric constant of the biomolecules in the cavity. The existence or nonexistence of the target biomolecule in the cavity modifies the dielectric constant of the dielectric where the cavity has been created, which changes the drain current of the device [1,2,3]. Though the dielectrically modulated tunnel-FET (TFET) based sensor has gathered the focus of researchers, as it reduces short-channel effect, TFET-based sensors suffer from high thermal budget requirements for source–drain formation and random dopant fluctuation arising from the difficulty in achieving the abrupt doping profile at source/drain-channel junctions [4]. Alternatively, Schottky barrier (SB) MOSFETs are considered as a prospective contender for high-performance CMOS ICs, as it is easy to form low-resistivity ultra-shallow junctions using metal source/drain instead of doped source/drain. Use of metal source/drain contact holds the advantage, as it commendably minimizes the issue of higher S/D series resistance and lessens the severe limitations imposed on conventionally implanted S/D. Further, intrinsic Schottky potential barrier of SB-MOSFETs results in greater control of OFF-state leakage current, and the subthreshold slope (SS) of SB-MOSFET has a lower limit of 60 mV/dec at room temperature [5,6,7,8,9,10]. Features such as low thermal budget requirements, higher invulnerability to short-channel effects, low source/drain (S/D) parasitic resistances, sub-10 nm gate length scalability and simple fabrication steps make Schottky barrier MOSFETs (SB-MOSFETs) a suitable FET biosensor for the detection of different biomolecules [3,4]. Nevertheless, SB-MOSFETs have many advantages compared to conventional MOSFETs. The intrinsic Schottky barrier between metal S/D and the semiconductor results in lower drive current [11,12,13]. Many SB devices, like dual metal gate, source/drain pocket doping, work function engineering, and plasma-based structures have been suggested in recent years to resolve the difficulties of SB-MOSFET, including ambipolar conduction and lower drive current [14,15,16,17,18,19,20]. Sumit Kale et al. employed dual-material S/D SB MOS with erbium silicide as the main S/D material and hafnium metal as the S/D extension material to suppress ambipolar leakage current in the SB-MOSFET [14]. Highly doped, dopant-segregated (DS) layers, which modulate the Schottky barrier (SB) height and width for improving the drive current of conventional SB-MOSFET, have been explored [15,16]. However, DS SB-MOSFET suffers from random dopant fluctuations (RDF) and increased thermal budget. Source engineered (SE) SB-MOSFET, using the charge-plasma concept to modify the SB width to eliminate RDF, has been studied. However, SE SBMOS suffer from ambipolar leakage current even for negative gate bias [17,18]. X Liu et al. have proposed the novel high-SB, bidirectional tunnel field effect transistor, which results in reduced thermionic emission and robust band-to-band (BTBT) forward tunneling current [10]. Sangeeta Singh et al. investigated the charge-plasma SB tunnel FET (CP-SB-TFET), and this study reveals that a pocket at both the drain and source end results in reduced ambipolar current, DIBL, and improved drive current [19]. Sumit Kale et al. have demonstrated that dopant segregation (DS) at the source-channel junction aids to increase tunneling area, which results in improved device performance with high ON current [20]. A ferroelectric SB tunnel FET (Fe SB-TFET) with a highly doped pocket at the source/drain and channel interface and gate–drain underlap reduce tunneling barrier width at the source side SB, resulting in improved device performance with low subthreshold swing (SS), reduced ambipolar current and high I_ON_/I_OFF_ [6]. Investigation of temperature’s effect on reliability issues of ferroelectric DS SB TFET reveals that the presence of a ferroelectric layer and the resulting negative capacitance effect increases the ON current, achieves highest I_ON_/I_OFF_ ratio and reduces the SS to 23 mV/dec at 300 K [21]. Silicon on insulator SB-MOSFET (SOI SB-MOSFET) with source extension (SE) and with source drain extension (SDE) significantly reduces drain-induced barrier tunneling and produces higher I_ON_/I_OFF_ and lower subthreshold swing (SS) than SOI SB-MOSFET [22].

The motivation of this work is to improve the performance of SB-MOSFET using structural modification and with source engineering. With this objective, a novel SB-MOSFET was made with quad gate structure and with SiGe at the source side of the channel. In this work, novel SB-MOSFET, named as quad gate SB-MOSFET, has been designed using TCAD, and its performance is analyzed using different electrical parameters such as ON current, I_ON_/I_OFF_ and subthreshold slope (SS). With this novel quad gate structure and source engineering, both I_ON_/I_OFF_ ratio and subthreshold slope have been improved. To substantiate, the performance improvement of the proposed quad gate SB-MOSFET’s electrical parameters is compared to the double gate SB-MOSFET. In this work, double gate SB-MOSFET is referred to as the conventional device as it has already been reported in the literature [23,24]. Further, the proposed device is used as a biosensor for detecting different biomolecules such as DNA, cellulose, APTES, biotin and streptavidin. This paper is organized as follows. The device structure and simulation approach are given in Section II, and simulation results of the proposed device and its comparison to the conventional device are presented in Section III. Application of the proposed device as a biosensor is given in Section IV, and the conclusion and future work are given in Section V.

## 2. Device Structure and Simulation Strategy

**Figure 1:**
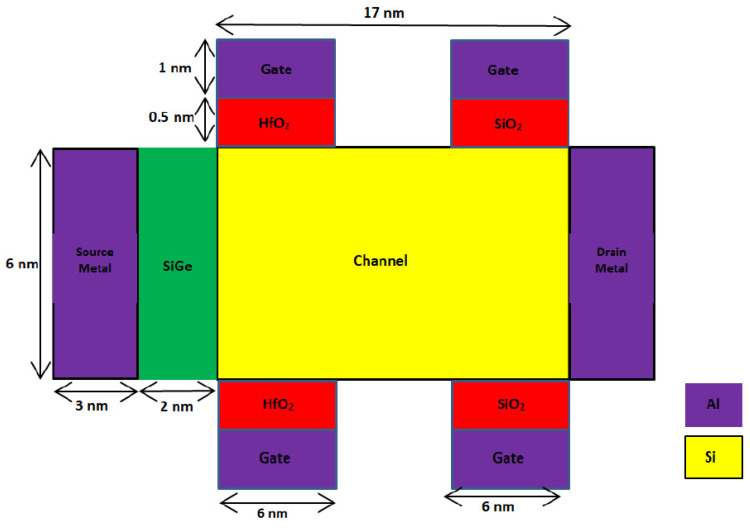
Schematic cross section of proposed quad gate SB-MOSFET.

**Figure 2:**
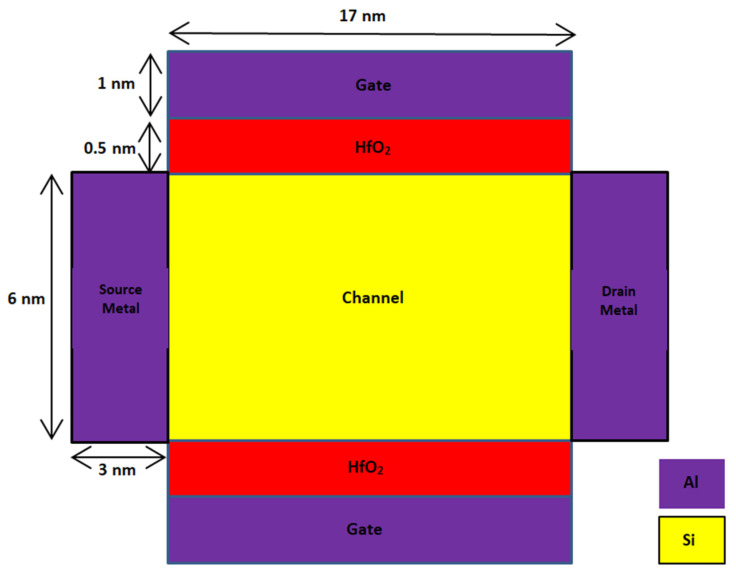
Schematic cross section of conventional double gate SB-MOSFET.

A 2D schematic cross section of the novel SB-MOSFET and conventional double gate SB-MOSFET are shown in Figure 1 and Figure 2, respectively. In both the devices, source is a heavily doped p-region with doping concentration of 10^20^ cm^−3^ and drain is n^+^ doped with a concentration of 10^18^ cm^−3^. The channel is p-type silicon with doping concentration of 10^15^ cm^−3^. Lower doping in the channel region is preferred, as it improves the carrier mobility, resulting in higher drain current. With the objective of designing a novel structure, two gate electrodes, with one gate dielectric at the source side and another at the drain side of the channel, have been used in this work. Both HfO_2_ and SiO_2_ are used as the gate dielectrics. The gate electrode, along with a high-κ dielectric (HfO_2_) at the source side of the channel, produces a higher electric field and enhances the tunneling rate, resulting in a higher ON-state current. Use of high-κ gate dielectric at the source side increases the internal electric field, and the high dielectric constant of HfO_2_ increases gate capacitance, resulting in a higher I_ON_/I_OFF_ ratio of the proposed device. Further, high-κ dielectric reduces the OFF-state leakage current due to direct tunneling, as it enables the use of a thicker gate oxide for the same gate capacitance, thereby reducing the power dissipation. The gate electrode at the drain side, along with SiO_2_ as gate dielectric, broadens the barrier at the drain/channel junction and prevents carrier tunneling, thereby suppressing the ambipolar current [24,25]. Aluminum with a work function of 4.1 eV is used as the metal contact. Aluminum at the source and drain, along with silicon substrate, form a Schottky contact. In the proposed device, the gate is not continuous throughout the channel and is present only at the source and drain side of the channel. The gate at the source side improves the tunneling, while the gate at the drain side reduces the leakage current. As there are four gate contacts, two at the top side and two at the bottom side of the channel, the name “quad gate” was given to the proposed SB-MOSFET. In this work, the gate structure is novel, i.e., the gate is present only at the source side and drain side of the channel. Further, to improve the carrier mobility, SiGe is introduced at the source side of the channel. Use of SiGe at the source side, and the resulting lower Schottky barrier, enhance the injection of the electron into the channel, thereby improving current drive capability. In contrast, a higher Schottky barrier for holes effectively suppresses hole injection into the silicon channel, thereby preventing the flow of holes in the OFF-state. Further, SiGe improves electron mobility by straining the crystal lattice, resulting in higher drive currents [26,27]. Silvaco TCAD is used for the design and simulation of the proposed device. The Universal Schottky Tunneling (UST) model captures tunneling close to the source channel junction while mobility models, viz., concentration mobility (CONMOB) and field dependent mobility (FLDMOB), have been used to capture different types of mobility. Band-gap narrowing models take into account the band-gap narrowing due to high doping concentrations of the source and drain while the Shockley–Read–Hall (SRH) model captures the effect of thermal generation leakage currents. Further, the transport mechanism in the device is simulated by the drift-diffusion model. Table 1 shows the parameters of different regions of both the proposed and conventional devices considered for simulation.

## 3. Result and Discussion

**Figure 3:**
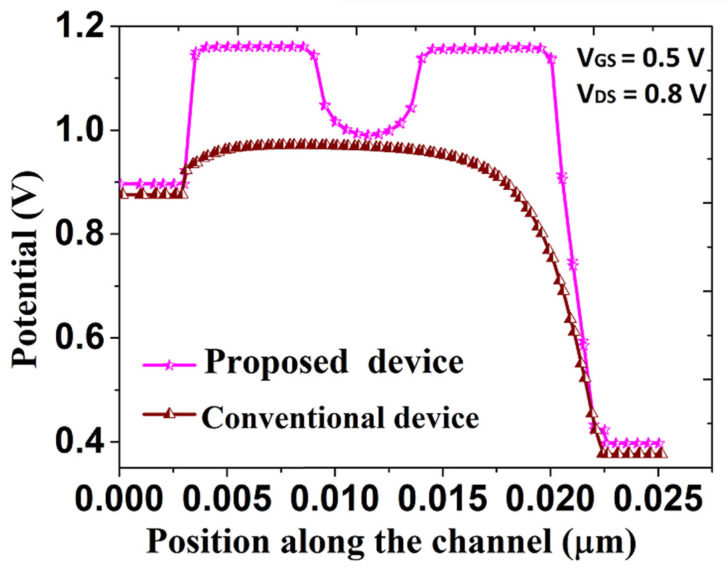
Variation of surface potential of proposed and conventional SB-MOSFET.

**Figure 4:**
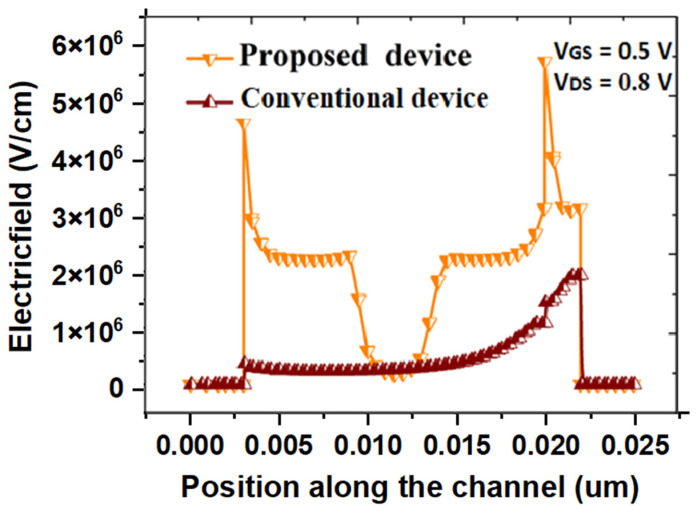
Variation of electric field of proposed and conventional SB-MOSFET.

**Figure 5:**
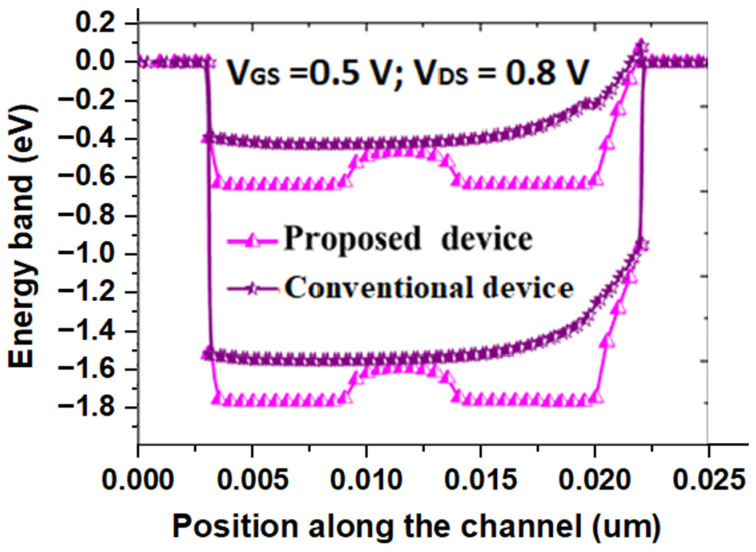
Energy band diagram of proposed and conventional SB-MOSFET.

**Figure 6:**
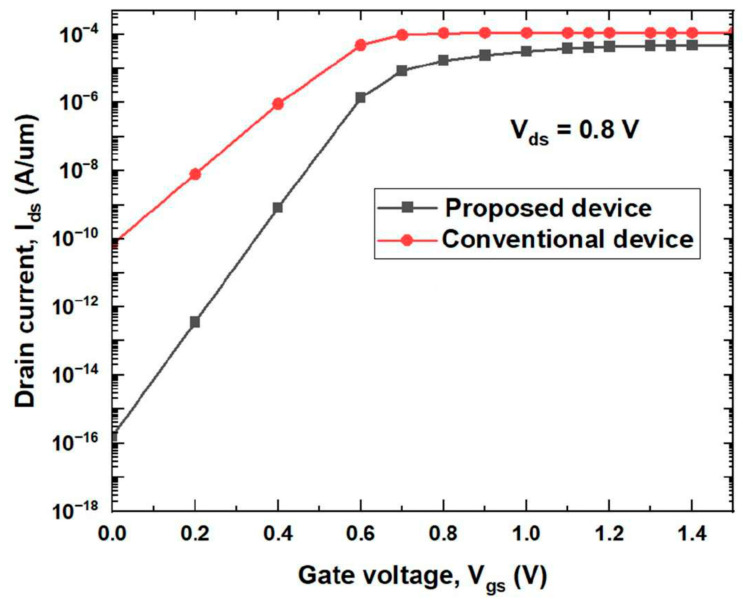
Comparison of transfer characteristics of proposed device with conventional device.

Different electrical characteristics, viz., surface potential, electric field, band energy and transfer characteristics of both the proposed and conventional DG SB-MOSFET, are analyzed and the corresponding results are presented in Figure 3, Figure 4, Figure 5 and Figure 6. It should be noted that in Figure 3, Figure 4 and Figure 5, the left end of the plot represents the drain side, while the right end corresponds to the source side of the channel. Surface potential variation along the channel length of both the proposed and conventional SB-MOSFET is presented in Figure 3. It has been observed that surface potential, a vital factor in estimating the DC property of thin-film transistor, is better in the proposed quad gate SB-MOSFET than in the conventional SB-MOSFET. The surface potential is almost 50% higher for the proposed device than conventional device. The surface potential is 1.15 V for the proposed device while the value is 0.75 V for the conventional device at 0.0215 μm for the bias voltage of V_gs_ = 0.5 V and V_ds_ = 0.8 V. Figure 4 presents variation of the electric field across the channel of both devices. It has been observed that the proposed quad gate SB-MOSFET produces a higher electric field at both the source and drain side of the channel than the conventional SB-MOSFET. The electric field is approximately three times higher in the quad gate SB-MOSFET than the conventional device. The electric field is 5.7 MV/cm in the proposed device while the value is 1.9 MV/cm in the conventional device. This higher electric field results in higher tunneling current, as BTBT generation rate is a strong function of the electric field. It can also be observed that in the proposed device, the electric field at the source/channel junction is higher than that value at the drain/channel junction. This could be attributed to the effect of the different gate electrodes at the source and drain side of the channel. HfO_2_ at the source side of the channel produces a higher electric field, which is useful for achieving a higher tunneling rate. The lower electric field resulting from the use of SiO_2_ as a gate dielectric at the drain side of the channel is useful for inhibiting tunneling of charges in the ambipolar state.

Figure 5 shows variation of the energy band along the channel length of both the SB-MOSFETs. The energy band diagram is measured at the gate–source voltage (V_GS_) of 0.5 V and drain–source voltage (V_DS_) of 0.8 V. It could be observed that the energy barrier width is significantly reduced at source side of the channel at the position of 0.0225 μm for the proposed quad gate SB-MOSFET than for the conventional device. The reason for SB thinning at the source side of the channel could be attributed to the use of a high-κ gate dielectric combined with the SiGe pocket region at the source side of the channel. A thinner energy barrier results in higher tunneling of electrons through the channel. Figure 6 presents drain current versus gate voltage characteristics of the proposed quad gate SB-MOSFETs compared to the conventional DG SB-MOSFET. It has been observed that there is a slight decrease in the ON current of quad gate SB-MOSFET; however, the proposed device exhibits a much lower OFF-state current. The OFF-state current of the proposed device is more than five orders lower than the conventional device. A significantly lower OFF-state current in the proposed device could be attributed to two reasons. The presence of the SiGe pocket region at the source side of the channel acts as an additional barrier in the OFF-state, ensuing in low leakage current. The other reason is aluminum, along with SiO_2_ at the drain side of the channel, broadens the drain/channel junction barrier, which results in a low tunneling probability of hole through the drain-side SB. It can also be observed that in the subthreshold region, there is a sharp decrease in drain current with respect to gate voltage in the proposed device compared to the conventional one, resulting in better subthreshold behavior of the proposed device. Electrical parameters of both the proposed and conventional DG SB-MOSFETs, calculated from the transfer characteristics, are compared and the results are given in Table 2. The proposed device exhibits superior performance with improved I_ON_/I_OFF_ ratio and reduced SS. The I_ON_/I_OFF_ ratio of the quad gate SB-MOSFET is five orders more than the conventional device while the subthreshold slope of quad SB-MOSFET is 25% lower than that of the conventional device. These results imply that the quad gate SB-MOSFET is suitable for future nano-scale ICs.

## 4. Applications

**Figure 7:**
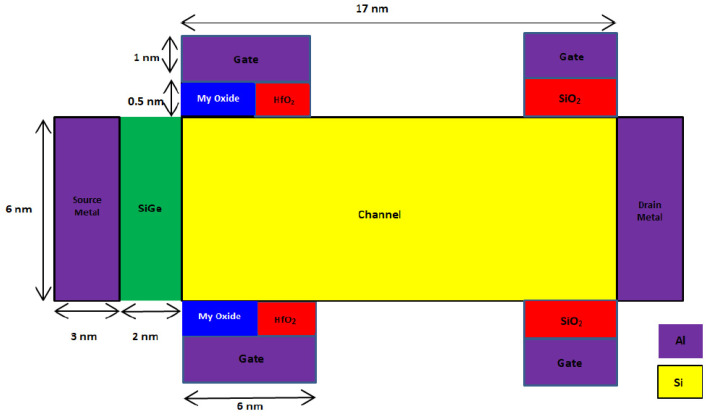
Schematic cross section of proposed quad gate SB-MOSFET Biosensor.

Biosensors have had a long journey, beginning from ion-sensitive FET [28] with a high sensitivity only to charged biomolecules like DNA, to present-day biosensors, which can detect neutral biomolecules like biotin and streptavidin. Biomolecules exist in different forms extending from nucleic acid, viruses, bacteria and proteins, and dimensions ranging from nm to μm. Knowledge of how these biomolecules function and their impact on several fields such as medicine, agriculture, and the food industry necessitated the early detection of biomolecules [29,30,31,32,33,34]. FET-based biosensors are realized by carving out a nanocavity at the source and/or drain end of the MOSFET. Existence of biomolecules in the nanocavity results in a change in the coupling between the gate and channel due to a change in dielectric constant of the gate oxide [34]. Further, this change in the dielectric constant results in SB thinning, resulting in higher tunneling current. Change in the drain current of the proposed quad gate SB-MOSFET biosensor can be used as an electrical parameter to detect the target biomolecules. The ability of the biosensor to detect the target biomolecules can be determined using the parameter of drain current sensitivity (*S_ID_*) and is given by [7]

(1) $$S_{I_{ON}}=\frac{I_{Bio}-I_{0}}{I_{0}}$$

where *I_Bio_* and *I*_0_ represent the ON-state current in the presence and absence of the biomolecules in the nanocavity, respectively. Higher sensitivity implies a higher chance of detecting the target species. Few works on FET-based biosensors have been reported in the literature [1,7]. S.A. Hafiz et al. have proposed source-engineered SB-FET, using the charge-plasma concept for sensing biomolecules, and observed that SE SB-FET exhibit much higher sensing capability for both neutral and charged biomolecules [1]. The L-shaped SB-FET biosensor designed with Al and Cu as a dual-material gate and with HfO_2_ as a gate dielectric exhibit better sensitivity at both low and high temperatures [7]. In this work, the proposed quad gate SB-MOSFET can be used as a biosensor for label-free detection of both charged and neutral biomolecules. The proposed device is converted into a biosensor by creating a nanocavity by etching gate oxide (HfO_2_) near the source junction of the channel. The size of the nanocavity is 4 nm × 0.5 nm. The proposed biosensor can be used for detecting different biomolecules such as streptavidin, biotin, APTES, cellulose and DNA which have unique dielectric constants, as given in Table 3. Biomolecules are placed in the nanocavity near the source end of the channel, and the corresponding variation in different electrical parameters such as surface potential, electric field and drain current are observed. The presence of different biomolecules in the nanocavity is modeled as oxide having different dielectric constants, as shown in Figure 7. To mirror the influence of a charged biomolecule, a fixed-interface oxide charge is included in the dielectric layer.

**Figure 8:**
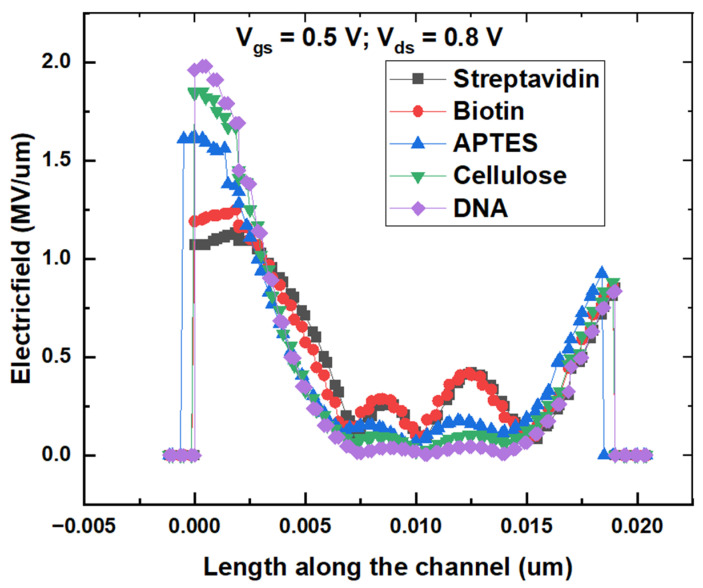
Electric field variation of proposed biosensor with different biomolecules.

Figure 8 shows the electric field variation across the channel with different biomolecules in the nanocavity. It can be seen that the electric field near the source end of the channel is highest for DNA and lowest for streptavidin, which concludes that the electric field increases with dielectric constant of the biomolecules. This could be attributed to the decrease in width of the depletion region due to thinning of the Schottky barrier, resulting in a higher electric field near the source.

**Figure 9:**
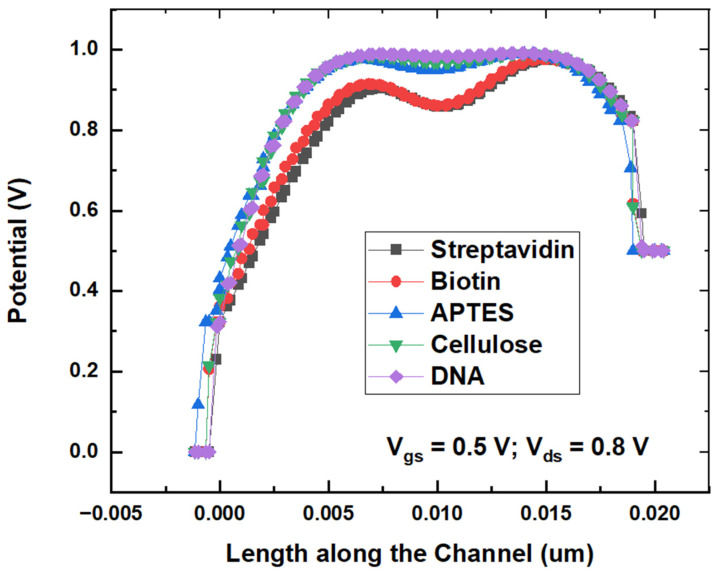
Surface potential variation for the proposed biosensor with different biomolecules.

Figure 9 shows the surface potential variation across the channel length with different biomolecules. It could be observed that potential is higher for biomolecules that have a higher dielectric constant, which could be ascribed to an increase in the capacitance with the dielectric constant of biomolecules in the cavity.

**Figure 10:**
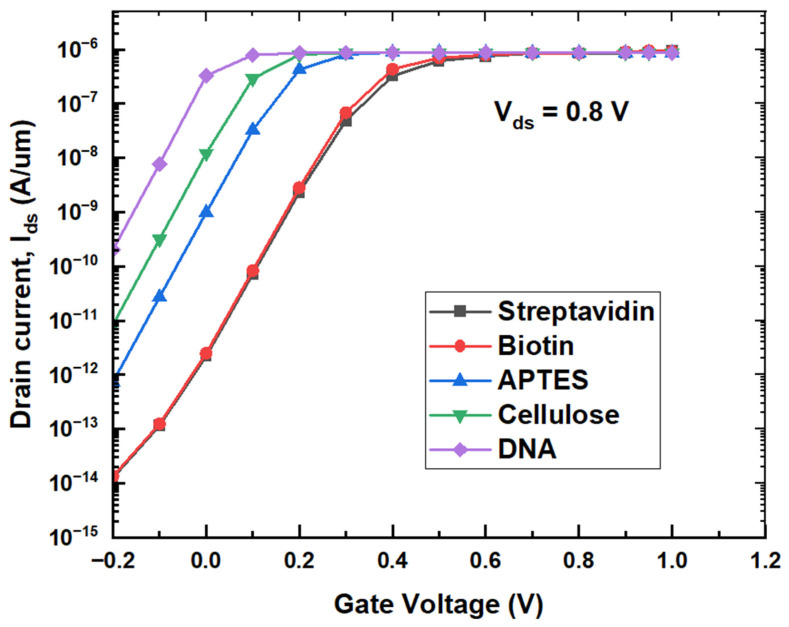
Transfer characteristics of proposed biosensor with different biomolecules.

**Figure 11:**
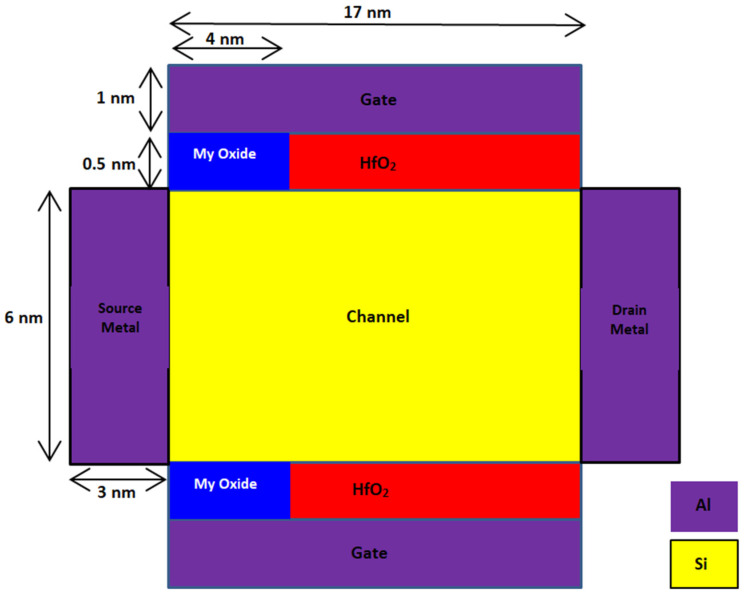
Schematic cross section of conventional DG SB-MOSFET Biosensor.

**Figure 12:**
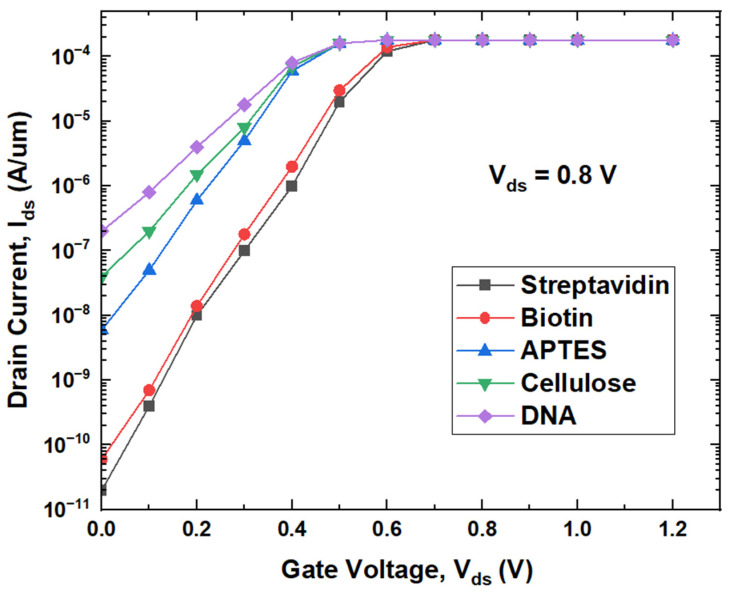
Transfer characteristics of conventional biosensor with different biomolecules.

It could be observed from Figure 10 that, for lower gate voltage, drain current increases with the dielectric constant, as DNA exhibits higher drain current while streptavidin produces lower drain current. An increase in the coupling capacitance with the dielectric constant results in higher charge concentration in the channel, resulting in an increase in drain current with the dielectric constant of the biomolecules. Surface potential variation along the length of the channel and transfer characteristics given in Figure 9 and Figure 10 are almost matching for both the biomolecules streptavidin and biotin. A small difference between the dielectric constants could be the reason behind almost-identical responses to these two biomolecules. To demonstrate the advantage of the proposed biosensor for the detection of different biomolecules, conventional biosensors are also designed by creating a nanocavity near the source side of the channel in the dielectric. Both the cross section and transfer characteristics of conventional biosensors are given in Figure 11 and Figure 12, respectively.

**Figure 13:**
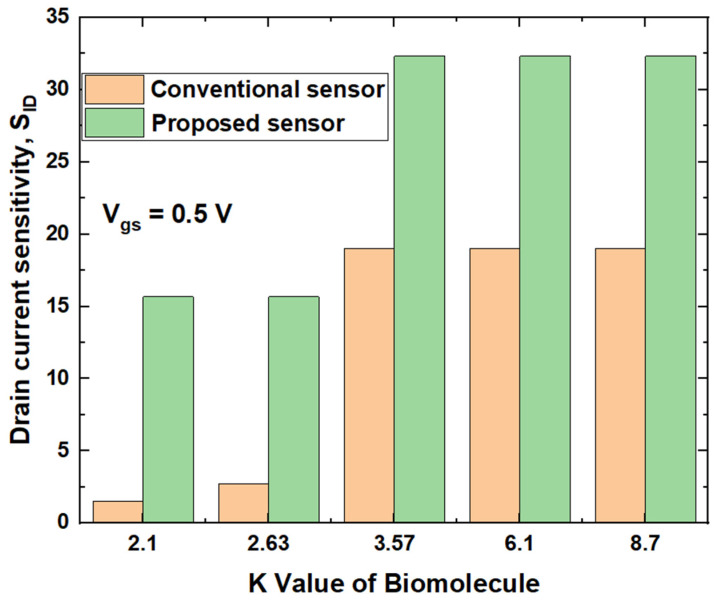
Comparison of drain current sensitivity of proposed and conventional biosensors.

Drain current sensitivity (S_ID_) of both the conventional and proposed biosensors are calculated by using the Equation (1) at the gate to source voltage of 0.5 V and given in Figure 13.

It can be observed that drain current sensitivity (S_ID_) increases with the increase in the dielectric constant, and the change is better in the proposed device than the conventional sensor made from DG SB-MOSFET. This could be ascribed to enhanced SB thinning resulting in a higher ON-current of the proposed device. Further, the S_ID_ value of biomolecules such as APTES, cellulose and DNA of the proposed sensor is found to be twice that of streptavidin and biotin. The S_ID_ value of DNA is 32.33 while the value of biotin is 15.67. This sensitivity analysis implies that the proposed biosensor outperforms the conventional sensor in the detection of all the five molecules and sensitivity is higher for biomolecules having a dielectric constant of more than 3.5. Selectivity is one of the vital parameters of the biosensor that determine how effectively the sensor detects the target biomolecule among the other biomolecules present in the cavity. Selectivity is calculated by taking the relative ratio of the drain current at different dielectric constant and is given by [31]

(2) $$\Delta S=\frac{I_{ON}\left(k=3.57,6.1,8.7\right)-I_{ON}\left(k=2.63\right)}{I_{ON}\left(k=2.63\right)}$$

**Figure 14:**
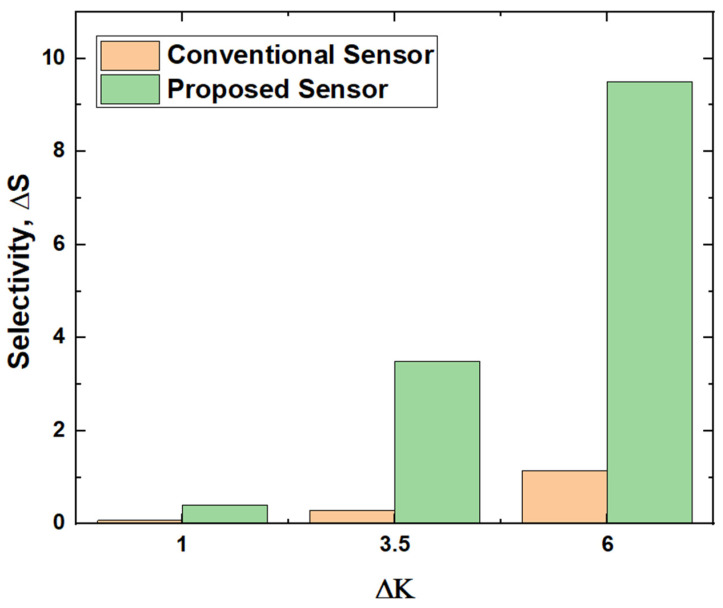
Selectivity of proposed and conventional biosensor.

Figure 14 presents the selectivity of biotin among the APTES, cellulose and DNA of both the proposed and conventional sensors. It could be observed that selectivity of the proposed sensor is six to twelve times higher than the conventional sensor based on the target biomolecules. 

## 5. Conclusions

In this paper, the novel SB-MOSFET-based biosensor for detecting the changes in physiological parameters of living organism has been proposed. The proposed device consists of a quad gate with a SiGe pocket region near the source end of the channel. Use of high-κ gate dielectric at the source end of the channel produces a higher electric field and enhances tunneling probability. On the other hand, SiO_2_ as the gate dielectric at the drain end broadens SB at the drain/channel junction, resulting in a reduced OFF-state current. The proposed device is found to be suitable for nano biosensors for detecting various biomolecules as it exhibits better electrical characteristics with a higher I_ON_-I_OFF_ ratio and lower subthreshold slope. The I_ON_-I_OFF_ ratio of the proposed device is six orders higher than the conventional double gate SB-MOSFET, and the subthreshold slope is 25% lower than that of the conventional device. Biosensors, both the proposed and conventional devices, are made by creating a nanocavity near the source end of the channel. It has been observed that drain current sensitivity increases with the dielectric constant of the biomolecules in the cavity. It is concluded that the proposed quad gate SB-MOSFET Biosensor has exceptional biosensing capability with higher sensitivity and selectivity than conventional bio sensors made from DG SB-MOSFET. In the future, further modification of the proposed device is required to distinguish biomolecules that have slightly different dielectric constants.

## Figures and Tables

**Table 1:** Simulation parameter of proposed quad gate and conventional DG SB-MOSFET.

Parameter	Quad Gate SB-MOSFET	Conventional SB-MOSFET
Gate oxide thickness	0.5 nm	0.5 nm
Silicon thickness	6 nm	6 nm
Source length	2 nm	2 nm
Doping concentration of Source	1020 cm^−3^	1020 cm^−3^
Doping concentration of drain	1018 cm^−3^	1018 cm^−3^
Channel length	17 nm	17 nm
Doping concentration of channel	1015 cm^−3^	1015 cm^−3^
Thickness of SiGe	6 nm	-
Length of SiGe	2 nm	-

**Table 2:** Comparison of electrical parameters of proposed and conventional SB-MOSFET.

Parameter	Quad Gate SB-MOSFET	Conventional SB-MOSFET
I_ON_	0.5 × 10^−4^	10^−4^
I_OFF_	1.5 × 10^−16^	9 × 10^−11^
I_ON_/I_OFF_	3.3 × 10^11^	1.1 × 10^6^
SS (mV/dec)	60.65	75.81

**Table 3:** Dielectric constant of biomolecules.

S.No	Biomolecules	Dielectric Constant
1	Streptavidin	2.1
2	Biotin	2.63
3	APTES	3.57
4	Cellulose	6.1
5	DNA	8.7

## Data Availability

Not applicable.
